# A review of the current anti-*Cryptococcus* antifungals and emerging treatment strategies

**DOI:** 10.1016/j.nmni.2026.101735

**Published:** 2026-03-03

**Authors:** Maphori Maliehe, Paulina M. Rapeso, Khwezi Mdana, Colleen K. McQuire, Lebogang Moukangwe, Nolwazi F. Ntshangase, Nozethu Mjokane, Zothile T. Skosana, Adepemi O. Ogundeji, Olufemi S. Folorunso, Carolina H. Pohl, Jacobus Albertyn, Olihile M. Sebolai

**Affiliations:** a Department of Microbiology and Biochemistry, University of the Free State, Bloemfontein, 9301, South Africa; b Universitas Academic Laboratory, National Health Laboratory Service, Bloemfontein, 9301, South Africa; c Department of Medical Microbiology, University of the Free State, Bloemfontein, 9301, South Africa

**Keywords:** Antifungal resistance, *Cryptococcus neoformans*, Drug repurposing, Immunomodulating therapies, Medicinal plants

## Abstract

Our knowledge of *Cryptococcus neoformans* and cryptococcal disease spans many years. Yet, despite numerous clinical studies and revisions to treatment protocols, the burden of cryptococcal disease remains alarmingly high, more so in endemic regions like Africa. Thus, the inclusion of this organism in the World Health Organization's (WHO) Fungal Priority Pathogens List (FPPL) in part underscores the unmet need to respond to cryptococcal infections and antifungal resistance adequately. To this end, this contribution interrogates the available literature, highlighting emerging treatment strategies to manage infections. Some of these strategies include drug repurposing, immunomodulating therapies and medicinal plants. Although some of the highlighted strategies are still only in the preclinical stages, these studies provide promising evidence of potential alternatives to currently available antifungals.

## Highlights

• *C. neoformans* has emerged as an important fungal pathogen of global importance.
• Suboptimal therapy and disease-related complications heighten clinical failure risk.
• We highlight promising approaches that may help to control cryptococcal disease.

## Graphical abstract

**Figure:**
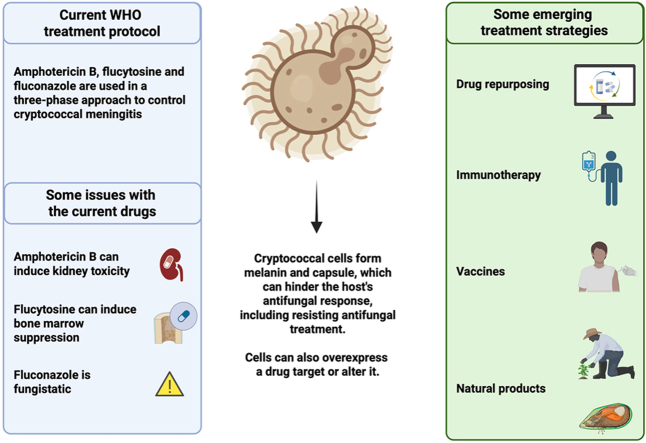

## 1 Introduction

*Cryptococcus (C.) neoformans* is a basidiomycetous yeast that rose from being an obscure terrestrial fungus to becoming a pathogen of global medical importance, with advanced HIV disease among adults being the main driver of cryptococcal infections [1,2]. The first description of cryptococcal disease was in 1894, after *C. neoformans* was isolated from a tibia lesion [3]. In Africa, it is documented that the first cases of cryptococcal meningitis surfaced around 1953 in the Congo River basin [4]. Therefore, we have known about cryptococcal disease for over 130 years, and in Africa for over 70 years. In the intervening period, significant strides have been made to understand the biology of *C. neoformans*. For example, the use of gene editing tools in elucidating the role of specific gene products in virulence [5], the delineation of *C. neoformans* into molecular types [6], the detection of cryptococcal capsular antigens, which later contributed to the development of the CrAg lateral flow assay as a point-of-care screening tool for the diagnosis of cryptococcal disease [7], establishing the antifungal sensitivity profiles of clinical isolates through the use of a universal standardised antimicrobial susceptibility testing protocol [8], and HIV case-control studies involving *C. neoformans* [9]. Despite all this knowledge, the continent remains disproportionately affected, carrying the highest burden of cases [[10], [11], [12]] compared to the rest of the world, with its people experiencing significant loss of lives and survivors left with higher disability-adjusted life years (DALYs) due to neurological defects induced by cryptococcal meningitis [13]. While there are efforts put in place to manage AIDS-related mycoses on the continent through the introduction of antifungals, the rise in drug resistance, which makes treatment sub-optimal, hampers efforts to impact the prevalence of cases significantly. Moreover, the management of patients is further exacerbated by complications associated with cryptococcal infections, such as elevated intracranial pressure, which, in some measure, may muddle patient management, leading to clinical failure. Thus, to some extent, the above explains the inclusion of *C. neoformans* in the critical group of the World Health Organisation's (WHO) fungal priority pathogens list [14].

Currently, five classes of antifungals are used to treat various fungal infections. These classes are polyenes (amphotericin B), azoles (fluconazole, itraconazole, voriconazole, isavuconazole and posaconazole), echinocandins (caspofungin, micafungin and anidulafungin), antimetabolites (flucytosine) and allylamines (terbinafine) [15]. However, only amphotericin B, fluconazole and flucytosine are recommended by the WHO for the treatment of cryptococcal infections [16]. This treatment generally occurs in a three-phase approach, consisting of the induction, consolidation, and maintenance phases. It is important to note that the drug regimen is combinatorial in nature, combining amphotericin B, flucytosine and fluconazole during the induction phase. Ideally, the paired drugs should display synergism. This is the desired outcome as it could help increase the spectrum of activity and minimise microbes' ability to exhibit non-antifungal susceptibility. The fluconazole and amphotericin B combination would see these two agents target the ergosterol, although at different levels. Therefore, by targeting the ergosterol, fluconazole reduces the target for amphotericin B. To be specific, fluconazole works by inhibiting ergosterol synthesis, leading to increased cellular permeability [17], while amphotericin B binds to ergosterol, creating pores and causing loss of intracellular content [18]. The idea that these two antifungal agents target ergosterol suggests there could be possible antagonism between them; however, this is not supported by experimental evidence obtained in laboratory animal studies [19,20] and patients enrolled in clinical trials [21], where results show reduced fungal burden and host survival.

On the other hand, the combination of amphotericin B and flucytosine is regarded as the “gold standard” and is more favoured for managing cryptococcal infections [22]. These two drugs are thought to act in synergy. This is because these two drugs have different targets, and the administration of amphotericin B, through damaging the cell membrane, may allow for increased uptake of flucytosine, which, in turn, disrupts DNA/RNA synthesis [23]. Importantly, their effectiveness in managing cryptococcal infections is supported by documented clinical evidence of increased rate of cerebrospinal fluid (CSF) sterilisation and decreased mortality [24]. Other studies considered pairing fluconazole and flucytosine. For example, Phase III of the ACTA trial found that the oral combination of fluconazole and flucytosine was non-inferior to the previous gold standard of combining amphotericin B and flucytosine in terms of 2-week mortality [25].

When reviewing the WHO guidelines, it is apparent that fluconazole is essential during all the phases of treatment for cryptococcal infections. This lengthy use of fluconazole is concerning! It was, thus, unsurprising when several studies in different parts of the world, *viz*. Taiwan [26], Uganda [27], South Africa [28] and China [29], have alluded to a rise in *C. neoformans* clinical isolates exhibiting fluconazole resistance. This observation led to the Southern African HIV Clinicians Society recommending higher fluconazole dosing in order to manage cryptococcal meningitis. For example, for induction, a fluconazole dose of 1200 mg per day was recommended versus the old dosage of 800 mg per day [30]. The same can be said about the rise in amphotericin B-non-susceptibility and flucytosine-non-susceptibility. This is because, before the worldwide spread of HIV/AIDS in the 1980s and the consequent rise in cryptococcal infections, the recommended course of treatment for cryptococcal infections was a daily dose of 0.3 mg/kg of amphotericin B [31], while today the dosage has been adjusted to 0.7 - 1 mg/kg of amphotericin B [16]. A summary of the evolution of cryptococcal meningitis treatment protocols is provided in Table 1.

**Table 1:** The evolution of cryptococcal meningitis treatment protocols.

Guideline document	Era and population group	Drug used during the induction phase; dosage in mg/kg/day			Citation
		Amphotericin B dose	Flucytosine dose	Fluconazole dose	
Bennett et al., 1979 guideline	Pre-HIV pandemic (immunocompetent patients)	0.3	150	0 (not on the market)	[31]
WHO 2011 guideline	During-HIV pandemic (immunocompromised population)	0.7-1.0	100	800	[149]
WHO 2018 guideline	During-HIV pandemic (immunocompromised population)	1.0	100	400-800	[150]
WHO 2022 guideline	During-HIV pandemic (immunocompromised population)	1.0	100	1200	[16]

Thus, on the whole, when considering the above, it can be reasoned that microbes will, as an inherent trait, adapt and withstand higher concentrations of antifungal drugs, thus continuing to threaten the efficacy of long-established standard dosing. Therefore, consideration should be given to other approaches to overcome treatment challenges associated with the management of cryptococcal infections, as the need for effective antifungals remains unmet. To this end, we have interrogated the available antifungal literature and highlight below some interventional measures that may prove useful in controlling cryptococcal infections and, by extension, fungi in general.

## 2 Some current clinical trials for improved management of associated cryptococcal meningitis

Recent clinical trial activity to improve induction therapy for HIV-associated cryptococcal meningitis has focused on strategies that shorten treatment and reduce toxicity. For example, a Phase II, the NCT06414512 open-label trial evaluated a reduced flucytosine dose, *i.e*., 60 mg/kg/day for 10 days, together with a single 10 mg/kg liposomal amphotericin B dose [32]. This study reported that reduced flucytosine dosage demonstrated inferior efficacy in CSF fungal clearance, raising concern that substantial dose reduction may compromise early mycological clearance [33]. Thus, these findings suggest that further investigation of dose optimisation is required. Related to this, a Phase I trial study registered as ISRCTN18180872 was designed to evaluate how a new sustained-release formulation of flucytosine behaves in the body among individuals with early cryptococcal disease [34]. At this point, patient-level efficacy data have yet to be published. The most recent advancement in cryptococcal meningitis research was the launch of the Platform Trial for Cryptococcal Meningitis, *i.e*., NCT06666322 [35]. This is a parallel programme initiated in 2025 by the University of Minnesota, and this trial introduces an innovative research framework designed to accelerate the identification of safer and more effective antifungal regimens for HIV-associated cryptococcal meningitis. The trial targets specifically low- and middle-income regions with a high HIV-associated cryptococcal meningitis burden, where current therapies remain constrained by toxicity, administration complexity and limited drug availability.

## 3 Repurposing other drugs as anti-cryptococcal antifungals

The previous section highlighted the challenge of rising incidents of reduced susceptibility towards antifungals as a major impediment to managing infections. A possible solution may be drug repurposing. The National Center for Advancing Translational Sciences defines drug repurposing as "studying drugs that are already approved to treat one disease or condition to see if they are safe and effective for treating other conditions" [36]. In the past, repurposing had been a largely unintentional process. A drug would be developed to treat a known and predicted target with the expectation that it would produce a desired or positive therapeutic outcome [[37], [38], [39]]. However, an unpredicted 'off-target' would be observed, often perceived as an undesirable or adverse therapeutic outcome. Upon further investigation of the drug's mode of action against the pathophysiological process of a specific condition, favourable therapeutic results would emerge, leading to the repurposing of the drug as a new indication [[40], [41], [42]]. Repurposing is often favoured because these drugs are already on the market, and thus, their safety and toxicity profiles are already known.

**Fig. 1:**
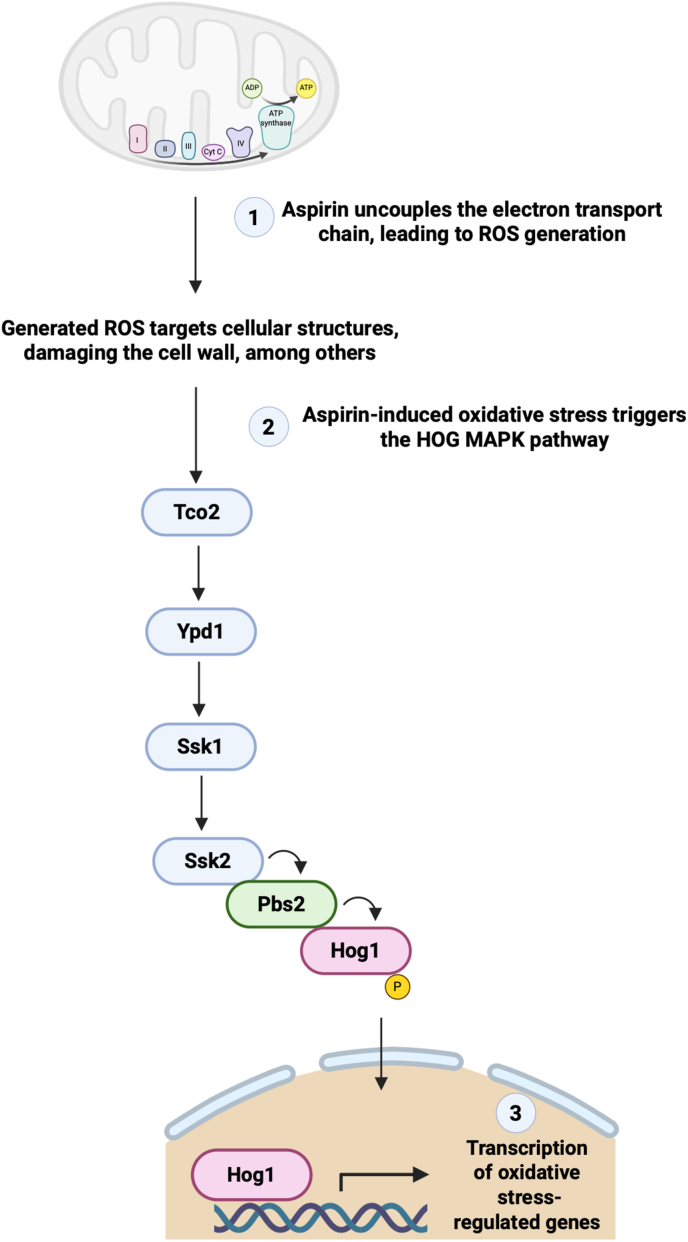
Mechanistic link between drug-induced ROS and fungal stress signalling. Exposure to aspirin or other ROS-inducing antifungal agents leads to the production of ROS, which results in membrane and cell wall damage. This oxidative stress activates the HOG MAPK pathway, which functions as a compensatory response to restore cellular homeostasis. This image was generated using Biorender.com.

Several drugs have been repurposed for the treatment of cryptococcal infection, and fortunately, some of these drugs have made it past the *in vitro* stage and into clinical trials. For example, Butts and co-workers screened a set of 1120 off-patent drugs for anti-cryptococcal activity [43]. In this work, they discovered 31 active compounds, including antipsychotic agents like phenothiazines, thioridazine, and trifluoperazine. As these drugs could cross the blood-brain barrier, they were considered beneficial for treating cryptococcal meningitis. Moreover, these co-workers also noted synergism between phenothiazine and fluconazole. In a subsequent study, Montoya et al. focused on optimising the antifungal properties of phenothiazine [44]. To this end, they performed structure-activity relationship analysis. They determined that the antifungal activity of phenothiazine correlated with the ability to inhibit calmodulin. This key regulatory protein is essential in the signalling pathway for fungal virulence and survival under host-imposed stress conditions [45]. A major concern with the use of these drugs is the potential neuroleptic side effects. Thus, it was encouraging that Montoya et al. observed that phenothiazine derivatives exhibited reduced affinity towards dopaminergic receptors, minimising undesirable neurological effects [44]. Ogundeji et al. demonstrated that aspirin inhibits cryptococcal cells by inducing the production of reactive oxygen species (ROS), which compromise membrane integrity and growth [46]. This ROS-mediated stress activates the HOG1 MAPK pathway, triggering a compensatory stress response that includes transcriptional adaptation. This study established a mechanistic link between drug-induced ROS and fungal stress signalling that may occur in other yeasts exposed to ROS-generating antifungal drugs (Fig. 1).

Other scholars have also studied the antifungal properties of sertraline, a selective serotonin reuptake inhibitor (SSRI) used to manage depression. The study by Zhai et al. illustrated both *in vitro* and *in vivo* antifungal activity against *C. neoformans* and that sertraline can act in synergy with fluconazole [47], while Trevinõ-Rangel et al. independently documented *in vitro* susceptibility and reduced fungal burden in the brain and spleen of mice [48]. While these results represented a possible new treatment option for managing cryptococcal infections, the observed *in vitro* success of sertraline did not translate into clinical success. This is highlighted in the works by Rhein and co-workers. These authors noted no significant improvement in clinical outcomes [49] and no mortality benefit [50]. However, after adopting a drug combination strategy, a desired therapeutic benefit could be realised. The latter is documented in the work of Katende et al., which sought to combine this drug with fluconazole and amphotericin B [51]. These authors noted that patients could tolerate the drug combination. A major conclusion from the study was the benefit of a short-course amphotericin B, which improved 10-week survival by 40%. Tamoxifen is another drug that has been repurposed against cryptococcal cells. It is a hormonal therapeutic agent that selectively blocks estrogen from binding to estrogen receptors, thus inhibiting the growth of cancerous cells [52]. In an *in vitro* study conducted by Dolan and co-workers, tamoxifen exhibited an inhibitory effect against *C*. *neoformans,* with treated cells showing an induced adenylate kinase release, indicating disruption to the cell membrane [53]. This drug was further shown to act in synergy with amphotericin B [54]. However, in a randomised study of 50 patients, Ngam et al. showed that even high doses of tamoxifen do not increase the rate of clearance of cryptococcal cells in the central nervous system (CNS) [55]. Antimalarial drugs have also been studied. For example, most recently, Wang et al. showed a synergistic relationship between hydroxychloroquine and itraconazole [56]. This work indicated that hydroxychloroquine treatment resulted in cell membrane damage and ruptured morphology of planktonic cryptococcal cells, which may have caused extracellular material leakage. Others have shown that antimalarial drugs can reduce fungal burden in a murine model [57,58]. In a recent study, Maliehe et al. evaluated the repurposing of the antimalarial artemisinin against *C. neoformans* and showed that the compound inhibits fungal growth by inducing mitochondrial dysfunction and cytochrome *c* release, consistent with apoptosis-like death [59]. This drug also enhanced fungal clearance by macrophages and improved larval survival in the *Galleria* (*G*.) *mellonella* model. Ma et al. reviewed the antifungal potential of the gold-based drug auranofin, which disrupts fungal redox balance by inhibiting thioredoxin reductase and mitochondrial protein import, leading to oxidative stress and growth inhibition [60].

From the above studies, it is apparent that despite the existence of promising preclinical results, clinical studies have yielded variable outcomes regarding the efficacy of some drugs. To this end, factors such as pharmacokinetics, optimal dosing, and potential side effects require further investigation to fully assess their clinical utility. The key antifungal findings related to these repurposing studies are summarised in Table 2.

**Table 2:** Select repurposed drugs with reported antifungal activity against *Cryptococcus neoformans*.

Compound/Class	Stage	Key Antifungal Finding(s)	References
Trifluoperazine (phenothiazine)	Preclinical: *in vitro* and murine studies	- *In vitro* activity - Synergism with fluconazole	[39]
Phenothiazine derivatives	Preclinical: *in vitro* studies	- Increased *in vitro* activity - Reduced neurological effects	[44]
Sertraline	Preclinical: *in vitro* and murine studies	- *In vitro* activity - Synergy with fluconazole - Reduced fungal burden in murine models	[47]
Sertraline	Preclinical: *in vitro* and murine studies	- *In vitro* activity - Reduced fungal burden in murine models	[48]
Sertraline (adjunct)	Clinical: phase II in HIV+ patients with cryptococcal meningitis	- Did not significantly improve clinical outcomes, but showed safety - Issues with drug levels in the CNS	[49]
Sertraline (adjunct)	Clinical: phase III randomised controlled trial	- Similar fungal clearance rates compared to placebo - No mortality benefit	[50]
Sertraline combined with fluconazole and a short course of amphotericin B	Clinical: observational study in Tanzania	- Combination therapy was feasible and well tolerated. This suggested potential for improved outcomes in resource-limited settings	[51]
Tamoxifen	Preclinical: *in vitro*	- Demonstrated *in vitro* activity against *Cryptococcus*	[53]
Tamoxifen	Preclinical: *in vitro*	- Demonstrated *in vitro* activity against *Cryptococcus* - Synergism with amphotericin B	[54]
Tamoxifen (high-dose adjunct)	Clinical: open-label randomised controlled trial	- High dosage did not increase the clearance rate of *Cryptococcus* from CSF - Caused drug-induced QTc prolongation	[55]
Hydroxychloroquine	Preclinical: *in vitro* and *G*. *mellonella* studies	- Exhibited antifungal activity alone - Synergy with itraconazole - Reduced fungal burden in *G*. *mellonella* larvae	[56]
Chloroquine	Preclinical: murine studies	- Decreased fungal burden in brain - Increased mouse survival rate	[57]
Artemisinin	Preclinical: *in vitro* and *G. mellonella* studies	- Demonstrated *in vitro* activity against *Cryptococcus* - Increased *G. mellonella* larvae survival	[59]
Auranofin	Preclinical: *in vitro*	- Demonstrated *in vitro* activity against *Cryptococcus*	[60]

## 4 Drugs that chemosensitise macrophages to kill internalised cryptococcal cells

An invading pathogen that localises in the lung epithelium would typically provoke an immune response that involves the release of pro-inflammatory cytokines by T helper 1 cells, which leads to the release of pro-inflammatory cytokines that are crucial for the classical M1 activation of macrophages to internalise and kill fungal cells via phagocytosis [[61], [62], [63]]. However, *C. neoformans* is one of the few pathogens that is well-equipped to evade macrophage immunoprocessing and manipulate these cells in a Trojan horse-like manner in order to reach distal organs [64,65]. For example, cells internalised in the phagosomes can evoke the participation of antioxidant enzymes to neutralise reactive oxygen species that are released by host immune cells [66,67]. Moreover, the acidic phagosomal environment does not impair cryptococcal replication [68,69]. From the above, it is clear that a growth control strategy to combat cryptococcal cells should extend to include limiting yeast survival inside macrophages.

**Fig. 2:**
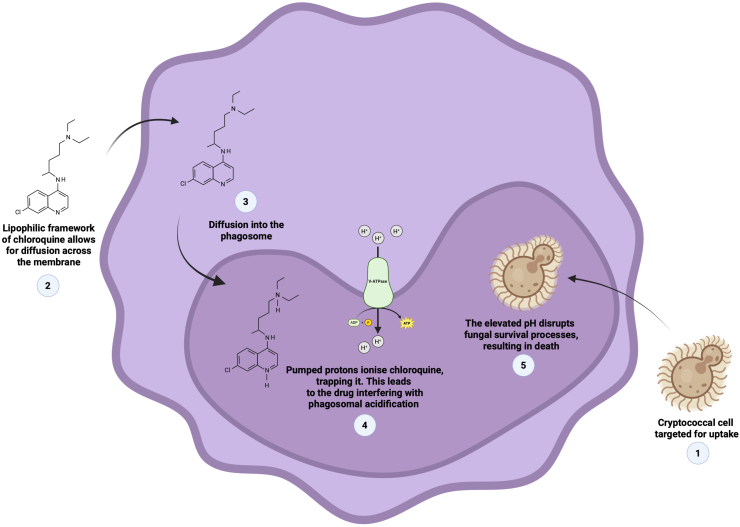
Drug-mediated disruption of the *Cryptococcus*-containing phagosome. A lipophilic drug like chloroquine may interfere with the acidic milieu of the phagosome harbouring a cryptococcal cell. The raised pH dismantles the specialised niche the fungus relies on for intracellular survival. This conceptual summary is based on the works of Madu et al. [70] and Levitz et al. [72]. This image was generated using Biorender.com.

Several studies have demonstrated the chemosensitisation of macrophages to improve their clearance of internalised cryptococcal cells. An important feature here is that a selected drug should be able to accumulate inside a macrophage to alter its internal environment. Madu and co-workers showed that primaquine-sensitised macrophages could significantly kill internalised cryptococcal cells [70,71]. Based on the chemistry of primaquine, *i.e.* that it is a lipophilic, weak base, it is possible that it may accumulate inside macrophages by ion trapping to, in turn, adjust the internal pH of macrophages. A similar observation was made by Levitz and co-workers when evaluating the effects of chloroquine on macrophages [72]. The raised pH may impair the proteolytic activity of enzymes [73] and limit the availability of intracellular iron nutrients crucial for growth [74]. Moreover, the raised pH also impairs the ability of cells to replicate, as cryptococcal cells are said to replicate optimally at a low pH, such as in the acidic phagosomes [75]. A pH-dependent killing mechanism is depicted in Fig. 2. The ability of primaquine to accumulate inside some organelles is not limited to macrophages. To this point, van Weert et al. documented the accumulation of primaquine inside endosomes [76]. It is conceivable that these drugs may be potentiating another killing method that is independent of pH neutralisation [76]. Here, it is possible that cells may be dying from direct contact with these drugs instead of an altered macrophage internal environment or enhanced macrophage phagocytosis.

In their work, Butts and co-workers showed that a derivative of phenothiazine, thioridazine, could accumulate within murine macrophages, in turn targeting and significantly reducing the intracellular burden of fungal cells, thus offering a potential therapeutic advantage [43]. Nosanchuk and co-workers also demonstrated that even the standard anti-cryptococcal drugs, fluconazole and amphotericin B, at sub-inhibitory concentrations, could enhance the phagocytosis of cryptococcal cells by macrophages. This led them to conclude that these drugs also exerted other subtle effects that contributed to their therapeutic efficacy [77]. A similar observation was noted when aspirin and ibuprofen were repurposed as anti-cryptococcal drugs. More to this point, Ogundeji et al. showed that aspirin and ibuprofen could enhance the ability of macrophages to kill internalised cryptococcal cells when compared to macrophages not exposed to these two anti-inflammatory drugs [46]. Moreover, the same authors further documented that when aspirin was functionalised as a ligand of a copper metal complex, it still retains its chemosensitising quality, resulting in increased phagocytosis of fungal cells [78]. The above studies showcase a nuanced approach aimed at amplifying the macrophage killing efficiency of intracellular pathogens like *Cryptococcus*. However, it is crucial also to avoid inducing a harmful inflammatory response that could become pathological instead of protective [79,80]. This is because macrophages also have cytotoxic effects on host tissue when not tightly regulated.

## 5 Other therapeutic interventions

### 5.1 Anti-virulence factor therapies

Compounds targeting virulence factors should be considered to complement the action of anti-*Cryptococcus* drugs. Cryptococcal virulence factors are arrays of cellularly produced functional biomolecules that promote host colonisation or suppress the host immune response to cause a diseased state [81]. The production of these factors is under the control of regulatory mechanisms, implying that interference with these regulatory mechanisms could affect their production [82,83]. In addition, knowing the biosynthetic pathway(s) that are involved in the production of virulence factors is equally important, as enzymes that catalyse these pathways may be targeted to modulate their activity [84].

Given the above scenarios, compounds could be used to disrupt gene expression or cause post-translational modifications to catalytic enzymes. In the end, the approach should attenuate the pathogenicity of the fungus. *Cryptococcus* has several key virulence factors that can be targeted, such as melanin [85]. This biomolecule overlays the cell wall and contributes to resilient cells that can resist host attack. To illustrate this point, Wang et al. [86] investigated the role of melanin in protecting cryptococcal cells against oxidative damage within macrophages and deduced that melanised cells were more resistant to killing by murine macrophages compared to non-melanised cells. At the same time, van Duin et al. [87] explored how melanisation affected the susceptibility of cells towards antifungal treatment. These authors noted that melanised cells exhibited decreased susceptibility towards amphotericin B and caspofungin but not drugs like fluconazole. Given the importance of this factor, its biosynthetic route is an ideal target for drug development. The laccase enzyme has been reported to be a key enzyme in the synthesis of melanin [88,89]. Thus, it was unsurprising that the deletion of the gene encoding for laccase led to reduced virulence of *C. neoformans* in *in vivo* studies [88,89]. This glycosylated copper protein enzyme synthesises melanin in several oxidation and reduction steps of several diphenolic substrates that consist of *para*- and *ortho*-diphenols, L-dopa, monophenols, and esculin, obtained extracellularly [90,91]. The inhibition of laccase may present with ancillary benefits. Fungal laccases can be inhibited by several compounds, such as L-cysteine [92]. These compounds have been shown to chelate the copper at the catalytic centre of laccase, thus disrupting the oxidation of a substrate and the subsequent transfer of electrons required to reduce oxygen [92,93]. In addition, compounds with a high affinity for binding pigments can be used. The work of Larsson [94] showed that trifluoperazine binds strongly to melanin, prolonging the drug's retention in the tissue. The latter may influence therapeutic efficacy, and this was an aspect that was explored by Wang and Casadevall [95]. In their paper, they evaluated trifluoperazine in the context of controlling *C*. *neoformans* and found that melanised cells were more susceptible. This contrasts with the general understanding that melanin shields microbes from antifungal stress [87], as reasoned by Ikeda et al. that melanin is a phenotypic antifungal resistance factor [96].

Another important pathogenic trait of cryptococcal cells is the ability to disseminate to the brain [97], and here, cells harness virulence factors such as proteases to breach the tightly regulated blood-brain barrier. More to this, Vu et al. showed that disseminating cells secreted a metalloprotease (Mpr1) that was essential for traversing the blood-brain barrier and establishing a cryptococcal infection in the CNS [98]. The latter was determined after showing that mutants that carry a deletion of the gene encoding Mpr1 could not breach an artificial blood-brain barrier model, while endogenous addition of recombinant Mpr1 promoted robust migration of yeast cells across the endothelium. Moreover, mammalian hosts infected with this mutant exhibited reduced brain fungal burden and improved survival. Together, these results highlighted Mpr1 as a possible target for developing a treatment option that disrupts dissemination to the brain. The latter was an aspect that Aaron et al. built upon by screening for natural products that could selectively block Mpr1 proteolysis [99]. In the end, they identified three lead compounds, abietic, diosgenin and lupinine that inhibited Mpr1 activity. Thus, these compounds were proposed as antivirulence drugs. As mammals lack Mpr1, the administration of these compounds would exclusively target fungal Mpr1 during the course of a developing cryptococcal disease.

#### 5.1.1 Ecological and molecular modulation of *C. neoformans* virulence

The ecological landscape of *C. neoformans* is shaped not only by its interactions with mammalian hosts but also by complex microbial consortia that inhabit shared environmental niches. Antagonism between bacteria and fungi has emerged as a critical modulator of cryptococcal physiology, influencing traits central to survival and virulence. The latter was highlighted by Mayer and Kronstad [100]. In their paper, these authors reported that diverse bacterial taxa such as *Pseudomonas* and *Staphylococcus* could inhibit the growth of cryptococcal cells through the secretion of secondary metabolites. However, the *Staphylococcus* mechanism is not defined. Among the best characterised examples of microbial antagonism is the interaction between *C. neoformans* and *Bacillus safensis*. To illustrate this, Mayer and Kronstad [101] reported that the species *Bacillus safensis* has anti-melanin and anti-capsular activity, which may involve the participation of chitinase upon cell-to-cell contact. Such antivirulence interactions suggest there is the potential to develop drugs that could attenuate pathogenicity while imposing minimal selective pressure for resistance. The above highlights that natural ecosystems can serve as reservoirs for antivirulence metabolites. It is equally important to note that some of these interactions, under specific conditions, were noted to promote cryptococcal virulence-related traits such as capsule and biofilm formation [102].

Some studies have highlighted mollusc-derived metabolites as promising bioactive agents against *C. neoformans* [103]. In this paper, the authors document that extracts from *Planorbella pilsbryi*, a freshwater mollusc, have an antifungal effect on cryptococcal cells comparable to fluconazole, while extracts from *Cipangopaludina chinensis*, a freshwater mollusc, affect cryptococcal thermotolerance and impair biofilm and capsule formation. Extracts from the land mollusc *Cepaea nemoralis* also impair capsule formation. These findings highlight interference with key cryptococcal virulence factors. Building on this, Gutiérrez-Góngora et al. showed that compounds from molluscs not only impair virulence traits but also impact fluconazole susceptibility in a clinical strain exposed to macrophages [104]. The latter shows the potential of using natural products as adjuncts to support current drugs. However, more studies are required to elucidate the mechanism of action of such compounds. At an intracellular level, virulence regulation is orchestrated through complex signalling networks. To this end, in their paper, Gutiérrez-Góngora et al. proposed that capsule impairment was potentially mediated through inhibition of cysteine peptidases involved in the Rim pathway [104]. A previous study by Geddes et al. used quantitative proteomics to define changes in the protein complement that occur upon modulating the cAMP signalling pathway that regulates virulence in *C. neoformans*, implicated proteostasis in capsule formation, and thus it was identified as a potential drug target [105]. In sum, the papers of Gutiérrez-Góngora et al. [104] and Geddes et al. [105] illustrate how proteomic analysis could enable the identification of proteins linked to virulence regulation in cells, thus could help guide the discovery of antivirulence compounds.

### 5.2 Fungal vaccines

While several different types of vaccines are available for treating viral and bacterial infections, as of now, no fungal vaccines are approved for therapeutic use in humans. However, several fungal vaccine candidates in preclinical and clinical development are highlighted as promising avenues for controlling fungal infections.

Rella et al. first showed that the deletion of the sterylglucosidase gene (*SGL1*) resulted in the accumulation of steryl glucosides on the cell wall surface of cryptococcal cells, coating the capsule [106]. Importantly, these mutants were shown to be avirulent and could be cleared from the lungs in 2 weeks. In a follow-up investigation, work from the same laboratory established the immunoprotective properties of this cryptococcal strain with a deleted *sgl1* gene. These authors determined that the mutant strain conferred strong protection in mice against subsequent wildtype or *C. gattii* challenge. Not only that, but also that the Δ*sgl1* vaccine strain was still protective even when given to immunocompromised mice lacking CD4^+^ T cells, CD8^+^ T cells, macrophages, monocytes, or neutrophils [[107], [108], [109]].

It is important to consider that while live attenuated vaccines may offer immunity, the primary concern is that in persons with a weakened immune system, the attenuated pathogen may still be capable of causing a disease [110]. To this end, subunit vaccines that employ purified antigens from the fungi to elicit an immune response may be a suitable alternative therapy as they pose no risk of infection [111]. These subunit vaccines typically require adjuncts in order to improve immunogenicity. These vaccines generally utilise components related to the cell wall and, thus, tend to be safer, especially in immunocompromised individuals. Cryptococcal cells are also known to be poorly immunogenic due to the polysaccharide capsule that masks the antigens on the cell wall and typically do not elicit a strong immune response when unconjugated [112,113]. Thus, when conjugated, galactoxylomannan (GalXM), a component of the capsule, elicits a robust and sustained antibody response in immunised mice [113]. In 1997, Zhang et al. laid the foundation for a mimotope-based vaccine strategy to control *C. neoformans* by identifying P13, a mimotope that mimics the capsular glucuronoxylomannan (GXM) [114]. This was further developed by Fleuridor et al. In their work, they show that raised antibodies could mediate fungal clearance, suggesting the mimotope may substitute for the carbohydrate-based antigen [115]. Datta and co-workers further built on this, showing the therapeutic efficacy of a P13-protein conjugated vaccine in a murine cryptococcosis model [116]. In summary, these works on P13 highlight the utility of epitome-mimicry in overcoming the challenge of polysaccharide-based vaccine design. As pointed out earlier, the capsule is poorly immunogenic in its native form.

In *Cryptococcus*, chitin deacetylases (CDAs) convert chitin to chitosan, the deacetylated form of chitin. Baker and co-workers reported on the importance of chitosan in maintaining cell wall integrity and retaining their melanin production phenotype, as the quadruple mutation *cda1cda2cda3fpd1* completely abolished the production of chitosan, rendering these strains non-pathogenic in mice [117,118]. This knowledge led to the development of alkaline extracts from these mutants packaged as glucan particle-based vaccines. These vaccines conferred protection and elicited a strong Th1- and Th17-biased CD4^+^ T cell response in infected mice [119]. This observation was supported by Upadhya et al., who reported that the *cda1Δ, cda2Δ,* and *cda3Δ* strains were avirulent in mice models and showed increased clearance from lungs due to production of proinflammatory molecules that mediate a Th1-type adaptive immune response [120]. Hester et al. further expanded on the pool of these protective protein antigens to develop more GP-based vaccines [121]. Further investigation indicated Cda2 to be a major stimulatory antigen when compared to Cda1 and Cda3. Immunoinformatic analysis of Cda2 predicted a peptide sequence that may bind strongly to the major histocompatibility complex class II (MHC II) H2-IAd allele in mice. This Cda2-Pep1 showed robust protection in mice [122]. Most recently, Li et al. utilised mRNA packaged in lipid nanoparticles (LNPs) to transcribe *CDA1* and showed that vaccination with *CDA1*-LNPs conferred protection in mice [123].

There are several studies that focused on developing a DNA vaccine related to the cryptococcal chitin deacetylase 2 (Cda2). Deletion of the gene encoding Cda2 resulted in a strain that was unable to produce chitosan. The study determined that vaccinating mice with a Cda2-encoding plasmid DNA coincided with a protective immune response, reduced fungal burden and prolonged survival following a challenge study [124]. This was further expanded on by Specht and co-workers using glucan particles, from *Saccharomyces cerevisiae*, as a delivery system for the Cda2 antigen [122,125,126]. The study determined that glucan particle-delivered Cda2 provided strong protection in mice, which was associated with reduced fungal burden and improved survival.

Table 3 summarises the above body of work on sterylglucosides (lipids), chitin deacetylase (when presented in a glucan particle), the P13 mimotope (peptide mimic), and the cryptococcal capsular polysaccharide GalXM (carbohydrate), and importantly underscores an emerging consensus that targeting non-protein components, *i.e*., lipids, carbohydrates, and mimotopes, can offer effective and immunogenic strategies for cryptococcal vaccine development.

**Table 3:** Comparative summary of experimental cryptococcal fungal vaccines.

Vaccine	Type	Antigen	Adjuvant	Target pathogen(s)	Development stage	References
*Δsgl1* mutant	Live-attenuated	Sterylglucoside-accumulating mutant (*Δsgl1*)	None (self-adjuvanting)	*C. neoformans;C. gattii*	Preclinical	[[107], [108], [109]]
Cda2 subunit vaccine	Subunit vaccine	Chitin deacetylase 2 (Cda2)	Glucan particles	*C. neoformans*	Preclinical	[124]
P13 mimotope vaccine	Subunit vaccine	Synthetic peptide mimicking GXM	Freund's adjuvant	*C. neoformans*	Preclinical	[114]
GalXM–protein conjugate	Subunit vaccine	GalXM conjugated to BSA	Freund's, Quil A	*C. neoformans*	Preclinical	[113]

### 5.3 Immunotherapy

The immune status of a host is important in determining the fate of invading pathogenic cells, *i.e*., infection clearance, persistent infection that is latent for an extended period or disseminated infection. Thus, the functioning of the immune system as an intact unit, wherein the interaction of innate cells with invading pathogens can lead to secondary immunological development, cannot be underestimated [61,62,127]. The latter is even more important in subjects who may have HIV, who, over time, progressively lose their adaptive immunity [b, [128], a]. Therefore, finding medicines that can modulate the functioning of the immune system may prove important in clearing infections. Some of these immunotherapies are discussed below.

Collins and Bancroft investigated how tumour necrosis factor-alpha (TNF-α) and granulocyte-macrophage colony-stimulating factor (GM-CSF) influence macrophage-mediated phagocytosis of cryptococcal cells [b, [128], a]. These authors determined that the combination significantly increased the uptake of cells by macrophages, suggesting that TNF-α could prime macrophages for more effective fungal clearance. Heung determined that TNF-α was essential for the classical activation of dendritic cells, thus could drive an adaptive immune response against fungal cells [129]. On the other hand, Fa and co-workers explored the effects of a mutant cryptococcal strain that produces TNF-α in a murine model of pulmonary cryptococcosis [130]. The authors demonstrated that the infected animals exhibited enhanced T-cell accumulation and a favourable Th1/Th2 cytokine balance. These studies underscore the essential role TNF-α plays in enhancing macrophage phagocytic activity to facilitate dendritic cell activation and T-cell-mediated immunity. Thus, TNF-α emerges as a critical effector molecule in antifungal defence to forestall the invading fungal cells.

The immunomodulatory properties of IFN-γ have been explored *in vivo* studies aimed at controlling a systemic cryptococcal infection in a murine model. Here, Kawakami and co-workers established that the neutralisation of endogenous IFN-γ by administering anti-IFN-γ monoclonal antibody (mAb) increased the number of fungal cells in the lung and brain while shortening the survival time of infected mice. The study further noted that the administration of IFN-γ decreased the number of fungal cells and extended the survival time of the mice [131]. This immunomodulatory quality was also explored in clinical studies. For example, a clinical trial study by Pappas and co-workers noted that the administration of a recombinant IFN-γ as adjunctive therapy led to clearance of the cryptococcal cells from the CNS, and importantly, patients did not display pronounced adverse effects [132]. As these promising findings warranted further investigation, it was encouraging to note a follow-up randomised controlled trial by Jarvis and co-workers evaluating the adjunctive qualities of IFN-γ in patients with HIV-associated cryptococcal meningitis [133]. This study determined that adding IFN-γ to standard antifungal treatment significantly accelerated the clearance of cryptococcal cells from the CSF.

Antibody treatment also offers a way to treat infections by binding to specific antigens on the cell wall surface, in turn alerting the immune system to resolve invading fungal cells, and this quality is demonstrated by the works of Rosas et al.and Larsen et al. In brief, a preclinical study by Rosas et al. on the efficacy of immunisation using monoclonal antibodies that bind melanin showed that passive immunisation prolonged the survival of and reduced the fungal burden in *C*. *neoformans*-infected mice in comparison to controls [134], while a Phase I trial study by Larsen et al. targeting the capsule, evaluated the safety and pharmacokinetics of the anti-cryptococcal antibody 18B7 in HIV-infected patients who had previously been treated for cryptococcal meningitis [135]. The study concluded that this antibody was safe and had the potential as an adjunctive therapy, as it could reduce the levels of cryptococcal antigens in the serum.

Notwithstanding the argument by Antachopoulos and Walsh that there is still insufficient clinical data to make reliable recommendations when it comes to immunotherapies [136]. The above studies show some merit in using immunomodulatory molecules as valuable adjuncts to antifungal treatment, particularly in cases with high fungal burdens or slow response to standard therapy. Also, the use of antibodies and cytokines may find broad-spectrum application, as they may work across different fungal species by enhancing the general immune response.

### 5.4 Plant-based medicines

Data from the World Health Organisation (WHO) suggests that, even with the availability of modern medicines, a significant portion of the world's population still relies on traditional or complementary/alternative medicines for their primary healthcare needs [137,138]. Coupled to this, Western medicines are increasingly expensive and thus inaccessible to many in developing countries [139]. Thus, many studies document the *in vitro* antifungal activity of plant extracts. However, the safety profile of the extracts is poorly characterised, especially long-term use in immunocompromised patients with cryptococcosis. Also, the lack of pharmacokinetics data, which is essential for establishing optimal dosing and therapeutic windows, limits the application of extracts. For this reason, the following discussion is limited to some identified active compounds that were assessed in preclinical studies involving mice. A study involving the use of berberine from *Berberis* species showed that it was well tolerated in mice at therapeutic doses [140]. This biocompound was thought to inhibit fungal ergosterol synthesis and reduce fungal loads in the lungs, thus improving the survival rate. Another preclinical study showed that thymol from *Thymus vulgaris* could control cryptococcal cells by regulating multiple signalling pathways, including calcineurin, unfolded protein response, and HOG (high-osmolarity glycerol) MAPK (mitogen-activated protein kinase) pathways. [141], while da Silva et al. showed that curcumin from *Curcuma longa* was well tolerated by mice, reduced fungal burden and extended survival time [142].

As scientific evidence supporting the use of medicinal plants for cryptococcal infections is generally sparse, the following discussion highlights studies that reached the clinical trial stage; however, these were for the management of other fungi and not *Cryptococcus*. Thus, the absence of human clinical trials, especially concerning cryptococcal disease, means that the safety, efficacy, and pharmacokinetics of extracts and identified biomolecules from plants in humans remain unestablished. Work by Satchell et al. explored the use of tea tree oil to treat interdigital tinea pedis in a double-blinded study involving 158 patients [143]. Patients applied the oil twice daily to affected areas for 4 weeks and were reviewed after treatment. Mycological cure rate was assessed by culture of skin scrapings after 4 weeks, and they recorded the cure rate as 64% in the tea tree oil group compared to 31% in the placebo group [143]. In another study involving 100 patients, a lotion made from the plant *Artemisia sieberi* was tested for the treatment of pityriasis versicolor, a condition caused by *Malassezia furfur*. For comparison reasons, a lotion with clotrimazole was included. In the end, the *Artemisia sieberi* lotion was more effective than the clotrimazole lotion in the treatment of pityriasis versicolor, as the clinical mycological cure for *Artemisia sieberi* lotion after 4 weeks was 86% compared to 59% with clotrimazole lotion [144]. A randomised triple-blind study, involving 84 non-pregnant women with vulvovaginal candidiasis (VVC), showed that a vaginal cream composed of *Nigella sativa* and honey could reduce fungal culture results post-treatment. Specifically, 67.5% of the *Nigella savita* group were culture negative for *Candida*, while 74.4% of women who received clotrimazole cream were culture negative for *Candida.* Notably, both groups experienced an improvement in symptoms, *e.g*., reduction in secretion, redness and itching post-treatment, and no participant reported side effects in either group [145].

## 6 Conclusion and perspectives

The rise of mycotic infections in clinical settings is parallel to a rise in antifungal resistance. Unfortunately, only a limited class of antifungals are available to manage infections in part because of selective toxicity, as fungi and humans share similar cellular targets. In here, we highlighted the long-term use of fluconazole as a potential issue. At the same time, it is important to understand that drug resistance is a dynamic phenomenon and could arise from the environment, *i.e*., soil, wherein the possible use of azole fungicides in agriculture could create selective pressure on these environmental fungi. And these resistant strains could cause infections in persons without prior exposure to azoles. This is a point that Smith and workers noted when observing increasing fluconazole resistance in Ugandan cryptococcal strains [27]. Therefore, there is a need to limit the application of azoles in agriculture. Studies like that of Smith and co-workers are essential as they also report on the emergence of antifungal resistance in clinical settings and could be used to argue for the revision and establishment of new standard dosing.

We also highlighted current clinical trials aimed at optimising antifungal regimens to improve patient outcomes, including innovative emerging strategies to establish new antifungals, such as drug repurposing. While the idea of drug repurposing holds promise, it is possible that it may not always yield desired outcomes. To illustrate this point, the repurposing of sertraline, an antidepressant drug, as an anti-*Cryptococcus* drug showed encouraging results with *in vitro* and *in vivo* studies but failed in a Phase III clinical trial [47,48,50]. There are some exciting results involving immunomodulating therapies, whether through chemosensitising immune cells, administering cytokines/antibodies or using vaccines. Albeit some work is still in the preclinical phase, this avenue offers a new direction to manage infections in the future as drug resistance grows and the traditional drug development approach lags behind. This approach may, at this time, be limited by high production costs and potential immune-associated adverse effects, especially in immunocompromised persons.

To manage fungal infections effectively, there should be equity and access to antifungal therapy, especially in low-resourced settings. Encouragingly, there are concerted efforts from interest groups to coalesce and form strategic partnerships in cryptococcal research and fungal research in general, intended to improve patient outcomes. For example, the mycology practitioners in the African Union Member States have worked closely with organisations like the Global Action for Fungal Infections (GAFFI) and presented the Diagnostics Survey Report at the 2nd International Conference on Public Health in Africa in Rwanda 2022 [146,147]. Another is the Pan African Mycology Working Group, which held a meeting in Nairobi in 2023 with representatives from the International Society for Human and Animal Mycology (ISHAM), European Confederation of Medical Mycology (ECMM), Centers for Disease Control and Prevention (CDC) and GAFFI. This meeting called for stronger continental collaboration, enhanced fungal disease surveillance, and leveraging of qualified human resources for mycology training, collaborations and exchange, now regarded as the Nairobi Declaration [148].

## CRediT authorship contribution statement

**Maphori Maliehe:** Writing – review & editing, Writing – original draft. **Paulina M. Rapeso:** Writing – review & editing, Writing – original draft. **Khwezi Mdana:** Writing – review & editing. **Colleen K. McQuire:** Writing – review & editing. **Lebogang Moukangwe:** Writing – review & editing. **Nolwazi F. Ntshangase:** Writing – review & editing. **Nozethu Mjokane:** Writing – review & editing. **Zothile T. Skosana:** Writing – review & editing. **Adepemi O. Ogundeji:** Writing – review & editing. **Olufemi S. Folorunso:** Writing – review & editing. **Carolina H. Pohl:** Writing – review & editing, Supervision. **Jacobus Albertyn:** Writing – review & editing, Supervision. **Olihile M. Sebolai:** Writing – review & editing, Supervision, Resources, Conceptualization.

## Funding

Olihile M. Sebolai is supported by grants from the 10.13039/501100001321National Research Foundation of South Africa (grant no. 137965), the 10.13039/501100001323Poliomyelitis Research Foundation of South Africa (grant no. 23/78), and the 10.13039/501100000265Medical Research Council of South Africa (SIR grant). These funders had no role in study design, data collection and analysis, publication decision, or manuscript preparation.

## Declaration of competing interest

The authors declare the following financial interests/personal relationships which may be considered as potential competing interests: Olihile M. Sebolai reports administrative support, article publishing charges, and equipment, drugs, or supplies were provided by National Research Foundation of South Africa. Olihile M. Sebolai reports administrative support, article publishing charges, and equipment, drugs, or supplies were provided by South African Medical Research Council. Olihile M. Sebolai reports administrative support, article publishing charges, and equipment, drugs, or supplies were provided by Poliomyelitis Research Foundation. If there are other authors, they declare that they have no known competing financial interests or personal relationships that could have appeared to influence the work reported in this paper.
